# A study on vague graphs

**DOI:** 10.1186/s40064-016-2892-z

**Published:** 2016-08-02

**Authors:** Hossein Rashmanlou, Sovan Samanta, Madhumangal Pal, R. A. Borzooei

**Affiliations:** 1 Young Researchers and Elite Club, Central Tehran Branch, Islamic Azad University, Tehran, Iran; 2 Department of Mathematics, Indian Institute of Information Technology, Nagpur, 440006 India; 3 Department of Applied Mathematics with Oceanology and Computer Programming, Vidyasagar University, Midnapore, 721102 India; 4 Department of Mathematics, Shahid Beheshti University, Tehran, Iran

**Keywords:** Vague graph, Vague h-morphism, Highly irregular vague graph

## Abstract

The main purpose of this paper is to introduce the notion of vague h-morphism on vague graphs and regular vague graphs. The action of vague h-morphism on vague strong regular graphs are studied. Some elegant results on weak and co weak isomorphism are derived. Also, $\mu$-complement of highly irregular vague graphs are defined.

## Background

Gau and Buehrer (1993) proposed the concept of vague set in 1993, by replacing the value of an element in a set with a subinterval of [0, 1]. Namely, a true-membership function $t_{v}(x)$ and a false membership function $f_{v}(x)$ are used to describe the boundaries of the membership degree. The initial definition given by Kauffman (1973) of a fuzzy graph was based the fuzzy relation proposed by Zadeh. Later Rosenfeld (1975) introduced the fuzzy analogue of several basic graph-theoretic concepts. After that, Mordeson and Nair (2001) defined the concept of complement of fuzzy graph and studied some operations on fuzzy graphs. Sunitha and Vijayakumar (2002) studied some properties of complement on fuzzy graphs. Many classifications of fuzzy graphs can be found in Boorzooei et al. (2016a, b), Rashmanlou and Pal (2013a, b), Rashmanlou et al. (2015a, b, c), Pramanik et al. (2016a, b), Samanta and Pal (2011b, 2012b, 2015, 2016) and Samanta et al. (2014c, d). Recently, Samanta and Pal (2011a, 2012a, 2013, 2014a, 2015) and Samanta et al. (2014b) defined different types of fuzzy graphs and established some important properties. To extent the theory of fuzzy graphs, Akram et al. (2014) introduced vague hypergraphs. After that, Ramakrishna (2009) introduced the concept of vague graphs and studied some of their properties.

In this paper, we introduce the notion of vague h-morphism on vague graphs and study the action of vague h-morphism on vague strong regular graphs. We derive some elegant results on weak and co weak isomorphism. Also, we define $\mu$-complement of highly irregular vague graphs.

## Preliminaries

By a graph $G^{*}=(V,E)$, we mean a non-trivial, finite connected and undirected graph without loops or multiple edges. A fuzzy graph $G=(\sigma ,\mu )$ is a pair of functions $\sigma :V\rightarrow [0,1]$ and $\mu :V\times V\rightarrow [0,1]$ with $\mu (u,v)\le \sigma (u)\wedge \sigma (v)$, for all $u,v\in V$, where *V* is a finite non-empty set and $\wedge$ denote minimum.

### **Definition 1**

(Gau and Buehrer 1993) A vague set *A* on a set *X* is a pair $(t_A; f_A)$ where $t_A$ and $f_A$ are real valued functions defined on $X\rightarrow [0, 1]$, such that $t_A(x)+f_A(x)<1$ for all $x\in X$. The interval $[t_A(x), 1-f_A(x)]$ is called the vague value of *x* in *A*.

In the above definition, $t_{A}(x)$ is considered as the lower bound for degree of membership of *x* in *A* and $f_{A}(x)$ is the lower bound for negative of membership of *x* in *A*. So, the degree of membership of *x* in the vague set *A* is characterized by the interval $[t_{A}(x),1-f_{A}(x)]$.

The definition of intuitionistic fuzzy graphs is follows. Let *V* be a non-empty set. An *intuitionistic fuzzy set (IFS)* in *V* is represented by $(V,\mu ,\nu )$, where $\mu :V\rightarrow [0,1]$ and $\nu :V\rightarrow [0,1]$ are membership function and non-membership function respectively such that $0\le \mu (x)+\nu (x)\le 1$ for all $x\in V$. Though intuitionistic fuzzy sets and vague sets look similar, analytically vague sets are more appropriate when representing vague data. The difference between them is discussed below.

**Fig. 1:**
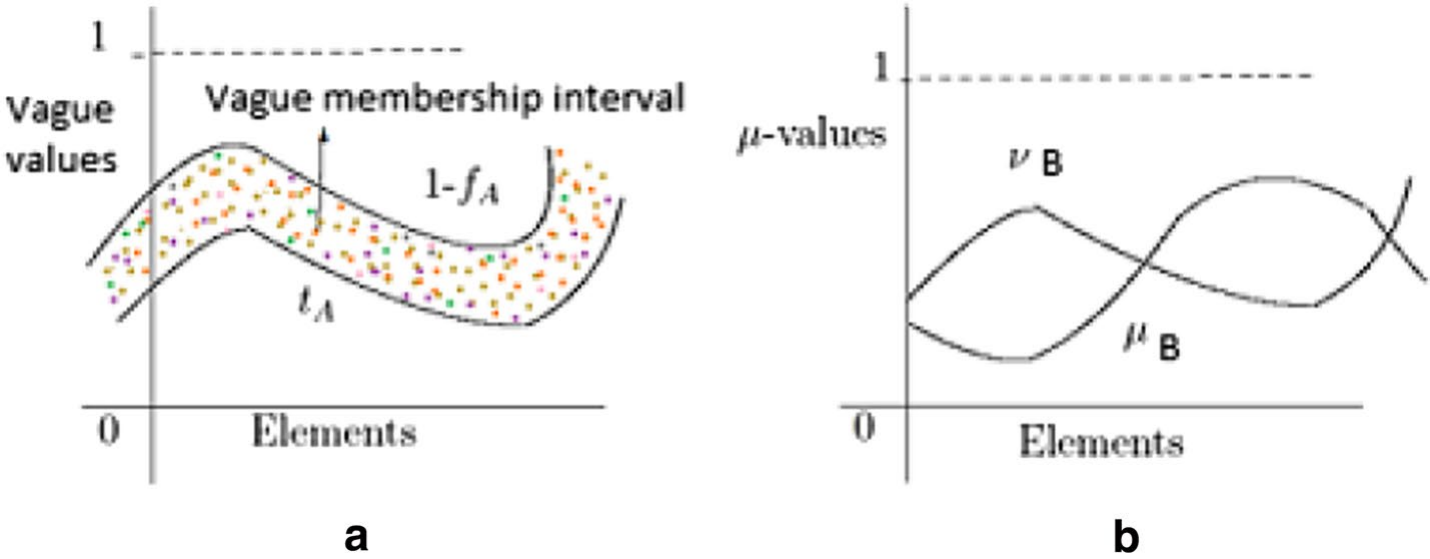
Comparison between **a** vague sets and **b** intutionistic fuzzy sets

The membership interval of an element *x* for vague set *A* is $[t_A(x),1-f_A(x)]$. But, the membership value for an element *y* in an intuitionistic fuzzy set *B* is $\langle \mu _B(y),\nu _B(y) \rangle$. Here, the semantics of $t_A$ is the same as with *A* and $\mu _B$ is the same as with *B*. However, the boundary is able to indicate the possible existence of a data value. This difference gives rise to a simpler but meaningful graphical view of data sets (see Fig. 1). It can be seen that, the shaded part formed by the boundary in a given Vague Set naturally represents the possible existence of data. Thus, this “hesitation region” corresponds to the intuition of representing vague data. We will see more benefits of using vague membership intervals in capturing data semantics in subsequent sections.

Let *X* and *Y* be ordinary finite non-empty sets. We call a vague relation to be a vague subset of $X\times Y$, that is an expression *R* defined by

$$\begin{aligned} R=\{\langle (x,y), t_{R}(x,y), f_{R}(x,y)\rangle \mid x\in X ,y\in Y\} \end{aligned}$$

where $t_{R}:X\times Y\rightarrow [0,1]$, $f_{R}:X\times Y\rightarrow [0,1]$, which satisfies the condition $0\le t_{R}(x,y)+f_{R}(x,y)\le 1$, for all $(x,y)\in X\times Y$. A vague relation *R* on *X* is called reflexive if $t_{R}(x,x)=1$ and $f_{R}(x,x)=0$ for all $x\in X$. A vague relation *R* is symmetric if $t_{R}(x,y)=t_{R}(y,x)$ and $f_{R}(x,y)=f_{R}(y,x)$, for all $x,y\in X$.

### **Definition 2**

Let $G^{*}=(V,E)$ be a crisp graph. A pair $G=(A,B)$ is called a vague graph on a crisp graph $G^{*}=(V,E)$, where $A=(t_{A},f_{A})$ is a vague set on *V* and $B=(t_{B},f_{B})$ is a vague set on $E\subseteq V\times V$ such that $t_{B}(xy)\le \min (t_{A}(x),t_{A}(y))\text{ and }\,\,f_{B}(xy)\ge \max (f_{A}(x),f_{A}(y))$ for each edge $xy\in E$. A vague graph *G* is called strong if $t_{B}(xy)=\min (t_{A}(x),t_{A}(y))\text{ and }\,\,f_{B}(xy)=\max (f_{A}(x),f_{A}(y))$ for all $x,y\in V$.

### **Definition 3**

The vague graph *G* is said to be regular if $\sum \nolimits _{v_{j},v_{i}\ne v_{j}}t_{B}(v_{i}v_{j})={\text{constant}}$ and $\sum \nolimits _{v_{j},v_{i}\ne v_{j}}f_{B}(v_{i}v_{j})={\text{constant}}$, for all $v_{i}\in V$. Moreover, it is called strong regular if

1.  $$t_{B}(v_{i}v_{j})=\min \{t_{A}(v_{i}),t_{A}(v_{j})\}$$ and $$f_{B}(v_{i}v_{j})=\max \{f_{A}(v_{i}),f_{A}(v_{j})\}$$.
2. $$\sum \nolimits _{v_{j},v_{i\ne v_{j}}}t_{B}(v_{i}v_{j})={\text{constant}}$$ and $$\sum \nolimits _{v_{j},v_{i\ne v_{j}}}f_{B}(v_{i}v_{j})={\text{constant}}$$.

### **Definition 4**

The complement of a vague graph $G=(A,B)$ is a vague graph $\overline{G}=(\overline{A},\overline{B})$ where $\overline{A}=A$ and $\overline{B}$ is described as follows. The true and false membership values for edges of $\overline{G}$ are given below.

$\overline{t_{B}}(uv)=t_{A}(u)\wedge t_{A}(v)-t_{B}(uv)$ and $\overline{f_{B}}(uv)=f_{B}(uv)-f_{A}(u)\vee f_{A}(v)$, for all $u,v\in V$.

### **Definition 5**

Let $G_{1}$ and $G_{2}$ be two vague graphs.

1. A homomorphism *h* from $G_{1}$ to $G_{2}$ is a mapping $h:V_{1}\rightarrow V_{2}$ which satisfies the following conditions:
  -  $$t_{A_{1}}(x_{1})\le t_{A_{2}}(h(x_{1})),\,\,f_{A_{1}}(x_{1})\ge f_{A_{2}}(h(x_{1}))$$,
  -  $$t_{B_{1}}(x_{1}y_{1})\le t_{B_{2}}(h(x_{1})h(y_{1}))$$, $$f_{B_{1}}(x_{1}y_{1})\ge f_{B_{2}}(h(x_{1})h(y_{1}))$$ for all $$x_{1}\in V_{1}$$, $$x_{1}y_{1}\in E_{1}$$.
2. An isomorphism *h* from $G_{1}$ to $G_{2}$ is a bijective mapping $h:V_{1}\rightarrow V_{2}$ which satisfies the following conditions:
  (c)  $$t_{A_{1}}(x_{1})=t_{A_{2}}(h(x_{1})),\,\,f_{A_{1}}(x_{1})=f_{A_{2}}(h(x_{1}))$$,
  (d) $$t_{B_{1}}(x_{1}y_{1})=t_{B_{2}}(h(x_{1})h(y_{1})),\,\,f_{B_{1}}(x_{1}y_{1})=f_{B_{2}}(h(x_{1})h(y_{1}))$$, for all $$x_{1}\in V_{1}$$, $$x_{1}y_{1}\in E_{1}$$.
3. A weak isomorphism *h* from $G_{1}$ to $G_{2}$ is a bijective mapping $h:V_{1}\rightarrow V_{2}$ which satisfies the following conditions:
  (e)  *h* is homomorphism,
  (f)  $t_{A_{1}}(x_{1})=t_{A_{2}}(h(x_{1})),\,\,f_{A_{1}}(x_{1})=f_{A_{2}}(h(x_{1}))$ for all $x_{1}\in V_{1}$. Thus a weak isomorphism preserves the weights of the nodes but not necessarily the weights of the arcs.
4. A co weak isomorphism *h* from $G_{1}$ to $G_{2}$ is a bijective mapping $h:V_{1}\rightarrow V_{2}$ which satisfies:
  (g)  *h* is homomorphism,
  (h)  $$t_{B_{1}}(x_{1}y_{1})=t_{B_{2}}(h(x_{1})h(y_{1})),\,\,f_{B_{1}}(x_{1}y_{1})=f_{B_{2}}(h(x_{1})h(y_{1}))$$, for all $$x_{1}y_{1}\in E_{1}$$.

### **Definition 6**

Let $G=(A,B)$ be a vague graph on $G^{*}$.

1. The open degree of a vertex *u* is defined as $\deg (u)=(d_{t}(u),d_{f}(u))$, where
  $$\begin{aligned} d_{t}(u)& = \mathop {\mathop {\sum }\limits _{u\ne v}}\limits _{v\in V}t_{B}(uv)\; {\text{and}}\\ d_{f}(u)& = \mathop {\mathop {\sum }\limits _{u\ne v}}\limits _{v\in V}f_{B}(uv). \end{aligned}$$
2. The order of *G* is defined and denoted as
  $$\begin{aligned} O(G)=\left( \sum _{u\in V}t_{A}(u),\sum _{u\in V}f_{A}(u)\right) . \end{aligned}$$
3. The size of *G* is defined as $S(G)=(S_{t}(G),S_{f}(G))= \left( \mathop {\mathop {\sum }\limits _{u\ne v}}\limits _{u,v\in V}t_{B}(uv),\mathop {\mathop {\sum }\limits _{u\ne v}}\limits _{u,v\in V }f_{B}(uv)\right)$.

## Regularity on isomorphic vague graph

In this section, we introduce the notion of vague h-morphism on vague graphs and regular vague graph. We derive some elegant results on weak and co weak isomorphisms.

### **Definition 7**

Let $$G_{1}$$ and $$G_{2}$$ be two vague graphs on $$(V_{1},E_{1})$$ and $$(V_{2},E_{2})$$, respectively. A bijective function $$h:V_{1}\rightarrow V_{2}$$ is called vague morphism or vague h-morphism if there exists positive numbers $$k_{1}$$ and $$k_{2}$$ such that $$(i)\,t_{A_{2}}(h(u))=k_{1}t_{A_{1}}(u)$$ and $$f_{A_{2}}(h(u))=k_{1}f_{A_{1}}(u)$$, for all $$u\in V_{1},$$$$(ii)\,t_{B_{2}}(h(u)h(v))=k_{2}t_{B_{1}}(uv)$$ and $$f_{B_{2}}(h(u)h(v))=k_{2}f_{B_{1}}(uv)$$, for all $$uv\in E_{1}$$. In such a case, *h* will be called a $$(k_{1},k_{2})$$ vague h-morphism from $$G_{1}$$ to $$G_{2}$$. If $$k_{1}=k_{2}=k$$, we call *h*, a vague k-morphism. When $$k=1$$ we obtain usual vague morphism.

### *Example 8*

**Fig. 2:**
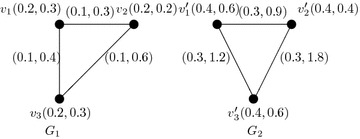
h-Morphism of vague graphs $G_1$ and $G_2$

Consider two vague graphs $G_1$ and $G_2$ defined as follows (see Fig. 2). Here, there is a vague *h*-morphism such that $h(v_1)=v'_1$, $h(v_2)=v'_2$, $h(v_3)=v'_3$, $k_1=2$, and $k_2=3$.

### **Theorem 9**

*The relation h-morphism is an equivalence relation in the collection of vague graphs*.

### *Proof*

Consider the collection of vague graphs. Define the relation $G_{1}\approx G_{2}$ if there exists a $(k_{1},k_{2})$ h-morphism from $G_{1}$ to $G_{2}$ where both $K_{1}$ and $K_{2}$ are non-zero. Consider the identity morphism from $G_{1}$ to $G_{1}$. It is a (1, 1) morphism from $G_{1}$ to $G_{1}$ and hence $\approx$ is reflexive.

Let $G_{1}\approx G_{2}$. Then there exists a $(k_{1},k_{2})$ morphism from $G_{1}$ to $G_{2}$, for some non-zero $k_{1}$ and $k_{2}$. Therefore $t_{A_{2}}(h(u))=k_{1}t_{A_{1}}(u)$, $f_{A_{2}}(h(u))=k_{1}f_{A_{1}}(u)$, for all $u\in V_{1}$ and $t_{B_{2}}(h(u)h(v))=k_{2}t_{B_{1}}(uv)$ and $f_{B_{2}}(h(u)h(v))=k_{2}f_{B_{1}}(uv)$, for all $uv\in E_{1}$. Consider $h^{-1}:G_{2}\rightarrow G_{1}$. Let $x,y\in V_{2}$. Since $h^{-1}$ is bijective, $x=h(u)$, $y=h(v)$, for some $u,v\in V_{2}$. Now, $t_{A_{1}}(h^{-1}(x))=t_{A_{1}}(h^{-1}(h(u)))=t_{A_{1}}(u)=\frac{1}{k_{1}}t_{A_{2}}(h(u))=\frac{1}{k_{1}}t_{A_{2}}(x)$. $f_{A_{1}}(h^{-1}(x))=f_{A_{1}}(h^{-1}(h(u)))=f_{A_{1}}(u)=\frac{1}{k_{1}}f_{A_{2}}(h(u))=\frac{1}{k_{1}}f_{A_{2}}(x)$. $t_{B_{1}}(h^{-1}(x)h^{-1}(y))=t_{B_{1}}(h^{-1}(h(u))h^{-1}(h(v)))=t_{B_{1}}(uv)=\frac{1}{k_{2}}t_{B_{2}}(h(u)h(v))=\frac{1}{k_{2}}t_{B_{2}}(xy)$, $t_{B_{1}}(h^{-1}(x)h^{-1}(y))=t_{B_{1}}(h^{-1}(h(u))h^{-1}(h(v)))=t_{B_{1}}(uv)=\frac{1}{k_{2}}t_{B_{2}}(h(u)h(v))=\frac{1}{k_{2}}t_{B_{2}}(xy)$, $f_{B_{1}}(h^{-1}(x)h^{-1}(y))=f_{B_{1}}(h^{-1}(h(u))h^{-1}(h(v)))=f_{B_{1}}(uv)=\frac{1}{k_{2}}f_{B_{2}}(h(u)h(v))=\frac{1}{k_{2}}f_{B_{2}}(xy)$. Thus there exists $\left( \frac{1}{k_{1}},\frac{1}{k_{2}}\right)$ morphism from $G_{2}$ to $G_{1}$. Therefore, $G_{2}\approx G_{1}$ and hence $\approx$ is symmetric.

Let $G_{1}\approx G_{2}$ and $G_{2}\approx G_{3}$. Then there exists a $(k_{1},k_{2})$ morphism from $G_{1}$ to $G_{2}$ say *h* for some non-zero $k_{1}$ and $k_{2}$ and there exists $(k_{3},k_{4})$ morphism from $G_{2}$ to $G_{3}$ say *g* for some non-zero $k_{3}$ and $k_{4}$. So, $t_{A_{3}}(g(x))=k_{3}t_{A_{2}}(x)$ and $f_{A_{3}}(g(x))=k_{3}f_{A_{2}}(x)$, for all $x\in V_{2}$ and $t_{B_{3}}(g(x)g(y))=k_{4}t_{B_{2}}(xy)$ and $f_{B_{3}}(g(x)g(y))=k_{4}f_{B_{2}}(xy)$, for all $xy\in E_{2}$. Let $f=g\circ h: G_{1}\rightarrow G_{3}$. Now,

$$\begin{aligned} t_{{A}_{3}}(f(u))= t_{A_{3}}((g\circ h)(u))& = t_{A_{3}}(g(h(u)))\\& = k_{3}t_{A_{3}}(h(u))\\& = k_{3}k_{1}t_{A_{1}}(u)\\ f_{{A}_{3}}(f(u))= f_{A_{3}}((g\circ h)(u))& = f_{A_{3}}(g(h(u)))\\& = k_{3}f_{A_{3}}(h(u))\\& = k_{3}k_{1}f_{A_{1}}(u)\\ t_{{B}_{3}}(f(u)f(v))= t_{{B_{3}}}((g\circ h)(u)(g\circ h)(v))& = t_{{B_{3}}}(g(h(u))g(h(v)))\\& = k_{4}t_{B_{2}}(h(u)h(v))\\& = k_{4}k_{2}t_{B_{1}}(uv)\\ f_{{B}_{3}}(f(u)f(v))= f_{B_{3}}((g\circ h)(u)(g\circ h)(v))& = f_{B_{3}}(g(h(u))g(h(v)))\\& = k_{4}f_{B_{2}}(h(u)h(v))\\& = k_{4}k_{2}f_{B_{1}}(uv). \end{aligned}$$

Thus there exists $(k_{3}k_{1},k_{4}k_{2})$ morphism *f* from $G_{1}$ to $G_{3}$. Therefore $G_{1}\approx G_{2}$ and hence $\approx$ is transitive. So, the relation h-morphism is an equivalence relation in the collection of vague graphs. $\square$

### **Theorem 10**

*Let*$G_{1}$*and*$G_{2}$*be two vague graphs such that*$G_{1}$*is*$(k_{1},k_{2})$*vague morphism to*$G_{2}$*for some non-zero*$k_{1}$*and*$k_{2}$. *The image of strong edge in*$G_{1}$*is strong edge in*$G_{2}$*if and only if*$k_{1}=k_{2}$.

### *Proof*

Let (*u*, *v*) be strong edge in $G_{1}$ such that *h*(*u*), *h*(*v*) is also strong edge in $G_{2}$. Now as $G_{1}\approx G_{2}$ we have

$$\begin{aligned} K_{2}t_{{B}_{1}}(uv)= t_{B_{2}}(h(u)h(v))& = t_{A_{2}}(h(u)\wedge h(v))\\& = k_{1}t_{A_{1}}(u)\wedge k_{1}t_{A_{1}}(v)\\& = k_{1}t_{B_{1}}(uv),\,\,\text{ for } \text{ all }\,\,u\in V_{1}. \end{aligned}$$

Hence, $k_{2}t_{B_{1}}(uv)=k_{1}t_{B_{1}}(uv)$, for all $u\in V_{1}$.

$$\begin{aligned} K_{2}f_{{B}_{1}}(uv)= f_{B_{2}}((h(u)h(v)))& = f_{A_{2}}(h(u)\vee h(v))\\& = k_{1}f_{A_{1}}(u)\vee k_{1}f_{A_{1}}(v)\\& = k_{1}f_{B_{1}}(uv),\,\,\text{ for } \text{ all }\,\,u\in V_{1}. \end{aligned}$$

Hence $k_{2}f_{B_{1}}(uv)=k_{1}f_{B_{1}}(uv)$, for all $u\in V_{1}$.

The equations holds if and only if $k_{1}=k_{2}$.□

### **Corollary 11**

*Let*$G_{1}$* and *$G_{2}$* be two vague graphs. Let*$G_{1}$* be*$(k_{1},k_{2})$* vague morphism to*$G_{2}$*. Let*$G_{1}$* be strong. Then*$G_{2}$* is strong if and only if*$k_{1}=k_{2}$.

### **Theorem 12**

*If a vague graphs*$G_{1}$*is co weak isomorphic to*$G_{2}$*and if*$G_{1}$*is regular then*$G_{2}$*is regular also.*

### *Proof*

As vague graph $G_{1}$ is co weak isomorphic to $G_{2}$, there exists a co weak isomorphism $h:G_{1}\rightarrow G_{2}$ which is bijective that satisfies $t_{A_{1}}(u)\le t_{A_{2}}(h(u))$ and $f_{A_{1}}(u)\ge f_{A_{2}}(h(u))$, $t_{B_{1}}(uv)=t_{B_{2}}(h(u)h(v))$ and $f_{B_{1}}(uv)=f_{B_{2}}(h(u)h(v))$, for all $u,v\in V_{1}$. As $G_{1}$ is regular, for $u\in V$, $\sum \nolimits _{u\ne v,v\in V_{1}}t_{B_{1}}(uv)=constant$ and $\sum \nolimits _{u\ne v,v\in V_{1}}f_{B_{1}}(uv)=constant$.

Now, $$\sum \nolimits _{h(u)\ne h(v)}t_{B_{2}}(h(u)h(v))$$$$=\sum \nolimits _{u\ne v,v\in V_{1}}t_{B_{1}}(uv)=constant$$ and $$\sum \nolimits _{h(u)\ne h(v)}f_{B_{2}}(h(u)h(v))$$$$=\sum _{u\ne v,v\in V_{1}}f_{B_{1}}(uv)=constant$$. Therefore $$G_{2}$$ is regular. $$\square$$

### **Corollary 13**

*If a vague graph*$G_{1}$*is co weak isomorphic to*$G_{2}$*and if*$G_{1}$*is strong, then*$G_{2}$*need not be strong*.

### **Theorem 14**

*Let*$G_{1}$*and*$G_{2}$*be two vague graphs. If*$G_{1}$*is weak isomorphic to*$G_{2}$*and if*$G_{1}$*is strong then*$G_{2}$*is strong also*.

### *Proof*

As vague graph $G_{1}$ is weak isomorphic with $G_{2}$, there exists a weak isomorphism $h:G_{1}\rightarrow G_{2}$ which is bijective that satisfies $t_{A_{1}}(u)=t_{A_{2}}(h(u))$, $f_{A_{1}}(u)=f_{A_{2}}(h(u))$, $t_{B_{1}}(uv)\le t_{B_{2}}(h(u)h(v))$ and $f_{B_{1}}(uv)\ge f_{B_{2}}(h(u)h(v))$. As $G_{1}$ is strong, $t_{B_{1}}(uv)=\min (t_{A_{1}}(u),t_{A_{1}}(v))$ and $f_{B_{1}}(uv)=\max (f_{A_{1}}(u)f_{A_{1}}(v))$. Now we have

$$\begin{aligned} t_{B_{2}}(h(u)h(v))\ge t_{B_{1}}(uv)&=\min (t_{A_{1}}(u),t_{A_{1}}(v))\\& = \min (t_{A_{2}}(h(u))t_{A_{2}}(h(v))). \end{aligned}$$

By the definition, $t_{B_{2}}(h(u)h(v))\le \min (t_{A_{2}}(h(u)),t_{A_{2}}(h(v)))$. Therefore, $t_{B_{2}}(h(u)h(v))=\min (t_{A_{2}}(h(u)),t_{A_{2}}(h(v)))$. Similarly,

$$\begin{aligned} f_{B_{2}}(h(u)h(v))\le f_{B_{1}}(uv)&=\max (f_{A_{1}}(u),f_{A_{1}}(v))\\& = \max (f_{A_{2}}(h(u))f_{A_{2}}(h(v))). \end{aligned}$$

By the definition, $\max (f_{A_{2}}(h(u)),f_{A_{2}}(h(v)))\le f_{B_{2}}(h(u)h(v))$. Therefore, $f_{B_{2}}(h(u)h(v))=\max (f_{A_{2}}(h(u)),f_{A_{2}}(h(v)))$. So, $G_{2}$ is strong. $\square$

### **Corollary 15**

*Let*$G_{1}$*and*$G_{2}$*be two vague graphs*. *If *$G_{1}$*is weak isomorphic to*$G_{2}$*and if*$G_{1}$*is regular, then*$G_{2}$*need not be regular*.

### **Theorem 16**

*If the vague graph*$G_{1}$*is co weak isomorphic with a strong regular vague graph*$G_{2}$, *then*$G_{1}$*is strong regular vague graph*.

### *Proof*

As vague graph $G_{1}$ is co weak isomorphic with a vague graph $G_{2}$, there exists a co weak isomorphism $h:G_{1}\rightarrow G_{2}$ which is bijective that satisfies $t_{A_{1}}(u)\le t_{A_{2}}(h(u))$, $f_{A_{1}}(u)\ge f_{A_{2}}(h(u))$, $t_{B_{1}}(uv)=t_{B_{2}}(h(u)h(v))$ and $f_{B_{1}}(uv)=f_{B_{2}}(h(u)h(v))$, for all $u,v\in V_{1}$. Now we have

$$\begin{aligned} t_{B_{1}}(uv)=t_{B_{2}}(h(u)h(v))& = \min (t_{A_{2}}(h(u)),t_{A_{2}}(h(v)))\\&\ge \min (t_{A_{1}}(u),t_{A_{1}}(v))\\ f_{B_{1}}(uv)=f_{B_{2}}(h(u)h(v))& = \max (f_{A_{2}}(h(u)),f_{A_{2}}(h(v)))\\&\le \max (f_{A_{1}}(u),f_{A_{1}}(v)). \end{aligned}$$

But by definition $t_{B_{1}}(uv)\le \min (t_{A_{1}}(u),t_{A_{1}}(v))$ and $f_{B_{1}}(uv)\ge \max (f_{A_{1}}(u),f_{A_{1}}(v))$. So, $t_{B_{1}}(uv)=\min (t_{A_{1}}(u),t_{A_{1}}(v))$ and $f_{B_{1}}(uv)=\max (f_{A_{1}}(u),f_{A_{1}}(v))$.

Therefore, $G_{1}$ is strong. Also for $u\in V_{1}$, $\sum \nolimits _{u\ne v, v\in V_{1}}t_{B_{1}}(uv)=\sum t_{B_{2}}(h(u)h(v))=constant$ as $G_{2}$ is regular and $\sum \nolimits _{u\ne v}f_{B_{1}}(uv)=\sum f_{B_{2}}(h(u)h(v))=constant$ as $G_{2}$ is regular. Therefore $G_{1}$ s regular. $\square$

### **Theorem 17**

*Let*$G_{1}$*and*$G_{2}$*be two isomorphic vague graphs, then*$G_{1}$*is strong regular if and only if*$G_{2}$*is strong regular*.

### *Proof*

As a vague graph $$G_{1}$$ s isomorphic with vague graph $$G_{2}$$, there exists an isomorphism $$h:G_{1}\rightarrow G_{2}$$ which is bijective and satisfies $$t_{A_{1}}(u)=t_{A_{2}}(h(u))$$ and $$f_{A_{1}}(u)=f_{A_{2}}(h(u))$$, for all $$u\in V_{1}$$ and $$t_{B_{1}}(uv)=t_{B_{2}}(h(u)h(v))$$ and $$f_{B_{1}}(uv)=f_{B_{2}}(h(u)h(v))$$, for all $$uv\in E_{1}$$. Now, $$G_{1}$$ is strong if and only if $$t_{B_{1}}(uv)=\min (t_{A_{1}}(u),t_{A_{1}}(v))$$ and $$f_{B_{1}}(uv)=\max (f_{A_{1}}(u),f_{A_{1}}(v))$$ if and only if $$t_{B_{2}}(h(u)h(v))=\min (t_{A_{2}}(h(u)),t_{A_{2}}(h(v)))$$ and $$f_{B_{2}}(h(u)h(v))$$$$=\max (f_{A_{2}}(h(u)),f_{A_{2}}(h(v)))$$ if and only if $$G_{2}$$ is strong. $$G_{1}$$ is regular if and only if $$\sum \nolimits _{u\ne v, v\in V_{1}}t_{B_{1}}(uv)=constant$$, for all $$u\in V_{1}$$ and $$\sum \nolimits _{u\ne v, v\in V_{1}}f_{B_{1}}(uv)=constant$$, for all $$u\in V_{1}$$ if and only if $$\sum \nolimits _{h(u)\ne h(v), h(v)\in V_{2}}t_{B_{2}}(h(u)h(v))=constant$$ and $$\sum \nolimits _{h(u)\ne h(v), h(v)\in V_{2}}f_{B_{2}}(h(u)h(v))=constant$$, for all $$h(u)\in V_{2}$$ if and only if $$G_{2}$$ is regular. $$\square$$

### **Theorem 18**

*A vague graphs*$G_{1}$*is strong regular if and only if its complement vague graph*$\overline{G}$*is strong regular vague graph also*.

### *Proof*

The complement of a vague graph is defines as $t_{A_{1}}=\overline{t_{A_{1}}}$, $f_{A_{1}}=\overline{f_{A_{1}}}$, $\overline{t_{B}}(uv)=t_{A}(u)\wedge t_{A}(v)-t_{B}(uv)$ and $\overline{f_{B}}(uv)=f_{B}(uv)-f_{A}(u)\vee f_{A}(v)$. *G* is strong regular if and only if $t_{B}(uv)=\min (t_{A}(u),t_{A}(v))$ and $f_{B}(uv)=\max (f_{A}(u),f_{A}(v))$ if and only if $\overline{t_{B}}(uv)=t_{A}(u)\wedge t_{A}(v)-t_{B}(uv)=t_{B}(uv)-t_{B}(uv)=0$ and $\overline{f_{B}}(uv)=f_{B}(uv)-f_{A}(u)\vee f_{A}(v)=f_{B}(uv)-f_{B}(uv)=0$ if and only if $\sum \overline{t_{B}}(uv)=0$ and $\sum \overline{f_{B}}(uv)=0$ if and only if $\overline{G}$ is strong regular vague graph. $\square$

### **Definition 19**

Let $G=(A,B)$ be a connected vague graph. *G* is said to be a highly irregular vague graph if every vertex of *G* is adjacent to vertices with distinct degrees.

### *Example 20*

**Fig. 3:**
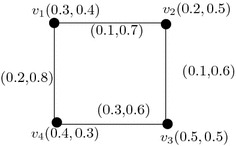
A highly irregular vague graph

Consider a vague graph *G* such that $V=\{v_{1},v_{2},v_{3},v_{4}\}$ and $E=\{v_{1}v_{2},v_{2}v_{3},v_{3}v_{4},v_{4}v_{1}\}$. By routine computations, we have $deg(v_{1})=(0.3,1.5)$, $deg(v_{2})=(0.2,1.3)$, $deg(v_{3})=(0.4,1.2)$, $deg(v_{4})=(0.5,1.4)$. We see that every vertex of *G* is adjacent to vertices with distinct degrees. So, *G* is highly irregular vague graph (see Fig. 3).

### **Theorem 21**

*For any two isomorphic highly irregular vague graphs, their order and size are same*.

### *Proof*

If *h* from $G_{1}$ to $G_{2}$ be an isomorphism between the highly irregular vague graphs $G_{1}$ and $G_{2}$ with the underlying sets $V_{1}$ and $V_{2}$ respectively then,

$$\begin{aligned} t_{A_{1}}(u)& = t_{A_{2}}(h(u)) , f_{A_{1}}(u)=f_{A_{2}}(h(u)),\,\,\text{ for } \text{ all }\,u\in V,\\ t_{B_{1}}(uv)& = t_{B_{2}}(h(u)h(v)) , f_{B_{1}}(uv)=f_{B_{2}}(h(u)h(v)),\,\,\text{ for } \text{ all }\,u,v\in V. \end{aligned}$$

So, we have

$$\begin{aligned} O(G_{1})& = \left( \sum _{u_{1}\in V_{1}}t_{A_{1}}(u_{1}),\sum _{u_{1}\in V_{1}}f_{A_{1}}(u_{1})\right) =\left( \sum _{u_{1}\in V_{1}}t_{A_{2}}(h(u_{1})),\sum _{u_{1}\in V_{1}}f_{A_{2}}(h(u_{1}))\right) \\& = \left( \sum _{u_{2}\in V_{2}}t_{A_{2}}(u_{2}),\sum _{u_{2}\in V_{2}}f_{A_{2}}(u_{2})\right) =O(G_{2})\\ S(G_{1})& = \left( \sum _{u_{1}v_{1}\in E_{1}}t_{B_{1}}(u_{1}v_{1}),\sum _{u_{1}v_{1}\in E_{1}}f_{B_{1}}(u_{1}v_{1})\right) \\& = \left( \sum _{u_{1},v_{1}\in V_{1}}t_{B_{2}}(h(u_{1})h(v_{1})),\sum _{u_{1},v_{1}\in V_{1}}f_{B_{2}}(h(u_{1})h(v_{1}))\right) \\& = \left( \sum _{u_{2}v_{2}\in E_{2}}t_{B_{2}}(u_{2}v_{2}),\sum _{u_{2}v_{2}\in E_{2}}f_{B_{2}}(u_{2}v_{2})\right) =S(G_{2}). \end{aligned}$$

$$\square$$

### **Theorem 22**

*If*$G_{1}$*and*$G_{2}$*are isomorphic highly irregular vague graphs then, the degrees of the corresponding verticesuandh*(*u*) *are preserved*.

### *Proof*

If $h:G_{1}\rightarrow G_{2}$ is an isomorphism between the highly irregular vague graphs $G_{1}$ and $G_{2}$ with the underlying sets $V_{1}$ and $V_{2}$ respectively then, $t_{B_{1}}(u_{1}v_{1})=t_{B_{2}}(h(u_{1})h(v_{1}))$ and $f_{B_{1}}(u_{1}v_{1})=f_{B_{2}}(h(u_{1})h(v_{1}))$ for all $u_{1},v_{1}\in V_{1}$. Therefore,

$$\begin{aligned} d_{t}(u_{1})& = \sum _{u_{1},v_{1}\in V_{1}}t_{B_1}(u_{1}v_{1})=\sum _{u_{1},v_{1}\in V_{1}}t_{B_2}(h(u_{1})h(v_{1}))=d_{t}(h(u_{1}))\\ d_{f}(u_{1})& = \sum _{u_{1},v_{1}\in V_{1}}f_{B_1}(u_{1}v_{1})=\sum _{u_{1},v_{1}\in V_{1}}f_{B_2}(h(u_{1})h(v_{1}))=d_{f}(h(u_{1})) \end{aligned}$$

for all $u_{1}\in V_{1}$. That is, the degrees of the corresponding vertices of $G_{1}$ and $G_{2}$ are the same. $\square$

### **Definition 23**

A vague graph *G* is said to be

1. self complementary if $G\cong \overline{G},$
2. self weak complementary if *G* is weak isomorphic with $\overline{G}$.

### Example 24

**Fig. 4:**
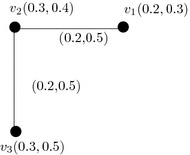
An example of vague graph with self complementary and weak complementary

Let us consider, a vague graph $G=(A,B)$ where, the vertex set be $V=\{v_1,v_2,v_2\}$ and edge set is $\{v_1v_2,v_2v_3\}$. Obviously, the graph is self complementary (see Fig. 4). If identity bijective mapping is assumed, then *G* and $\overline{G}$ are weak isomorphism.

### **Theorem 25**

*LetGbe a self weak complementary highly irregular vague graph then*,

$$\begin{aligned} \sum _{u\ne v}t_{B}(uv)&\le \frac{1}{2}\sum _{u\ne v}\min (t_{A}(u),t_{A}(v)),\\ \sum _{u\ne v}f_{B}(uv)&\ge \frac{1}{2}\sum _{u\ne v}\max (f_{A}(u),f_{A}(v)). \end{aligned}$$

### *Proof*

Let $G=(A,B)$ be a self weak complementary highly irregular vague graph of $G^{*}=(V,E)$. Then, there exists a weak isomorphism $h:G\rightarrow \overline{G}$ such that for all $u,v\in V$ we have $t_{A}(u)=\overline{t_{A}}(h(u))=t_{A}(h(u))$, $f_{A}(u)=\overline{f_{A}}(h(u))=f_{A}(h(u)) t_{B}(uv)\le \overline{t_{B}}(h(u)h(v))$, $f_{B}(uv)\ge \overline{f_{B}}(h(u)h(v))$.

Using the definition of complement in the above inequality, for all $u,v\in V$ we have

$$\begin{aligned} t_{B}(uv)\le \overline{t_{B}}(h(u)h(v))& = \min (t_{A}(h(u)),t_{A}h(v))-t_{B}(h(u)h(v))\\ f_{B}(uv)\ge \overline{f_{B}}(h(u)h(v))& = f_{B}(h(u)h(v))-\max (f_{A}(h(u)),f_{A}(h(v))\\ t_{B}(uv)+t_{B}(h(u)h(v))&\le \min (t_{A}(h(u)),t_{A}(h(v)))\\f_{B}(uv)+f_{B}(h(u)h(v))&\ge \max (f_{A}(h(u)),f_{A}(h(v))). \end{aligned}$$

So, $\sum \nolimits _{u\ne v}t_{B}(uv)+\sum \nolimits _{u\ne v}t_{B}(h(u)h(v))\le \sum \nolimits _{u\ne v}\min (t_{A}(h(u)),t_{A}(h(v)))$ and $\sum \nolimits _{u\ne v}f_{B}(uv)+\sum \nolimits _{u\ne v}f_{B}(h(u)h(v))\ge \sum \nolimits _{u\ne v}\max (f_{A}(h(u)),f_{A}(h(v)))$. Hence, $2 \sum \nolimits _{u\ne v}t_{B}(uv)\le \sum \nolimits _{u\ne v}\min (t_{A}(u),t_{A}(v))$ and $2 \sum \nolimits _{u\ne v}f_{B}(uv)\ge \sum \nolimits _{u\ne v}\max (f_{A}(u),f_{A}(v))$. Now we have $\sum \nolimits _{u\ne v}t_{B}(uv)\le \frac{1}{2}\sum \nolimits _{u\ne v}\min (t_{A}(u),t_{A}(v))$ and $\sum \nolimits _{u\ne v}f_{B}(uv)\ge \frac{1}{2}\sum \nolimits _{u\ne v}\max (f_{A}(u),f_{A}(v))$.

### **Definition 26**

Let $G=(A,B)$ be a vague graph. The $\mu$-complement of *G* is defined as $G^{\mu }=(A,B^{\mu })$ where $B^{\mu }=(t^{\mu }_{B},f_{B}^{\mu })$ and

$$\begin{aligned} t_{B}^{\mu }(uv)& = \left\{ \begin{array}{cc} t_{A}(u)\wedge t_{A}(v)-t_{B}(uv) &\quad \text{ if }\,t_{B}(uv)>0 \\ 0 &\quad \text{ if }\,t_{B}(uv)=0 \end{array}\right. \\ f_{B}^{\mu }(uv)& = \left\{ \begin{array}{cc} f_{B}(uv)-f_{A}(u)\vee f_{A}(v)&\quad \text{ if }\,f_{B}(uv)>0 \\ 0 &\quad \text{ if }\,f_{B}(uv)=0 \end{array}\right. \end{aligned}$$

### *Example 27*

**Fig. 5:**
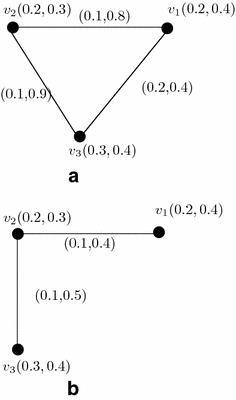
$\mu$-Complement of vague graphs. **a** Vague graph *G*. **b** *μ*-Complement of *G*

Let us consider a vague graph $G=(A,B)$ where the vertex set is $V=\{v_1,v_2,v_3\}$ and edge set is $E=\{v_1v_2,v_2v_3,v_1v_3\}$ (see Fig. 5).

### **Theorem 28**

*The*$\mu$-*complement of a highly irregular vague graph need not be highly irregular*.

### *Proof*

To every vertex, the adjacent vertices with distinct degrees or the non-adjacent vertices with distinct degrees may happen to be adjacent vertices with same degrees. This contradicts the definition of highly irregular vague graph. $\square$

### **Theorem 29**

*Let*$G_{1}$*and*$G_{2}$*be two highly irregular vague graphs. If*$G_{1}$*and*$G_{2}$*are isomorphic, then*$\mu$-*complement of*$G_{1}$*and*$G_{2}$*are isomorphic also and vice versa*.

### *Proof*

Assume that $G_{1}$ and $G_{2}$ are isomorphic, there exists a bijective map $h:V_{1}\rightarrow V_{2}$ satisfying $t_{A_{1}}(u)=t_{A_{2}}(h(u))$, $f_{A_{1}}(u)=f_{A_{2}}(h(u))$, for all $u\in V_{1}$ and $t_{B_{1}}(uv)=t_{B_{2}}(h(u)h(v))$, $f_{B_{1}}(uv)=f_{B_{2}}(h(u)h(v))$, for all $uv\in E_{1}$. By the definition of $\mu$-complement we have $t_{B_{1}}^{\mu }(uv)=\min (t_{A_{1}}(u),t_{A_{1}}(v))-t_{B_{1}}(uv)=\min (t_{A_{2}}(h(u)),t_{A_{2}}(h(v)))-t_{B_{2}}(h(u)h(v))$, $f_{B_{1}}^{\mu }(uv)=f_{B_{1}}(uv)-\max (f_{A_{1}}(u),f_{A_{1}}(v))=f_{B_{2}}(h(u)h(v))-\max (f_{A_{2}}(h(u)),f_{A_{2}}(h(v)))$, for all $uv\in E_{1}$. Hence, $G_{1}^{\mu }\cong G_{2}^{\mu }$. The proof of the converse part is straight forward. $\square$

## Conclusion

It is well known that graphs are among the most ubiquitous models of both natural and human-made structures. They can be used to model many types of relations and process dynamics in computer science, physical, biological and social systems. In this paper, we introduced the notion of vague h-morphism on vague graphs and studied the action of vague h-morphism on vague strong regular graphs. We defined $\mu$-complement of highly irregular vague graphs and investigated its properties.
